# Food Insecurity Among LGBQ+ Veterans

**DOI:** 10.1001/jamanetworkopen.2024.42979

**Published:** 2024-11-04

**Authors:** Sylvia V. Haigh, Christopher W. Halladay, Michael R. Kauth, Christine Going, Alicia J. Cohen

**Affiliations:** 1 Transformative Health Systems Research to Improve Veteran Equity and Independence Center of Innovation, Veterans Affairs Providence Healthcare System, Providence, Rhode Island; 2 LGBTQ+ Health Program, Veterans Health Administration, Department of Veterans Affairs, Washington, DC; 3 Department of Psychiatry, UMass Chan Medical School, Worcester, Massachusetts; 4 Food Security Office, Veterans Health Administration, Department of Veterans Affairs, Washington, DC; 5 Department of Family Medicine, Warren Alpert Medical School of Brown University, Providence, Rhode Island; 6 Department of Health Services, Policy, and Practice, Brown University School of Public Health, Providence, Rhode Island

## Abstract

**Importance:**

Food insecurity is associated with numerous adverse health outcomes. Little is known about the prevalence of and risks for food insecurity among veterans identifying as lesbian, gay, bisexual, queer, and similar (LGBQ+), a population facing unique social barriers and medical comorbidities.

**Objective:**

To examine food insecurity and potential risk factors among LGBQ+ veterans.

**Design, Setting, and Participants:**

This retrospective, cross-sectional study used administrative data from all US Veterans Health Administration (VHA) facilities nationally. Participants included veterans screened for food insecurity between March 1, 2021, and August 31, 2023.

**Exposure:**

Positive response to food insecurity screening administered in VHA facilities as part of routine clinical care.

**Main Outcomes and Measures:**

Prevalence of and sociodemographic, clinical, and psychosocial factors associated with food insecurity among veterans identifying as LGBQ+ or heterosexual and those with “don’t know” responses regarding their sexual orientation.

**Results:**

Of 3 580 148 veterans screened, the mean (SD) age was 61.6 (0.4) years; 3 192 507 (89.2%) were assigned male sex at birth. A total of 83 292 veterans (2.3%) identified as LGBQ+, and 10 183 (0.3%) had “don’t know” responses. LGBQ+ veterans (5352 [6.4%]) and veterans with “don’t know” responses (635 [6.2%]) were more than twice as likely as heterosexual veterans (90 426 [2.6%]) to have positive screen results for food insecurity. While risk factors for food insecurity were similar for veterans across sexual orientation groups, LGBQ+ veterans had higher rates of several risk factors compared with heterosexual veterans, including age younger than 45 years (45.3% vs 19.5%), female sex assigned at birth (44.1% vs 10.0%), being in a minoritized racial or ethnic group (34.7% vs 29.8%), unmarried or unpartnered status (69.1% vs 39.7%), low income (16.4% vs 14.9%), homelessness or housing instability (10.3% vs 5.4%), anxiety (7.7% vs 4.3%), depression (31.1% vs 19.3%), suicidality (3.6% vs 1.4%), posttraumatic stress disorder (42.2% vs 30.2%), substance use disorder (13.1% vs 9.0%), military sexual trauma (24.0% vs 5.4%), and recent intimate partner violence (2.6% vs 1.4%).

**Conclusions and Relevance:**

In this cohort study of veterans screened for food insecurity, LGBQ+ veterans and those with “don’t know” responses for sexual orientation experienced food insecurity at nearly 2.5 times the rate of heterosexual veterans. While risk factors for food insecurity were similar across groups, LGBQ+ veterans faced a higher prevalence of particular risks, including homelessness and several mental health and trauma-related comorbidities. Future work should examine targeted screening and interventions tailored to identifying and addressing food insecurity in this population, given their increased vulnerability and burden of food insecurity.

## Key Points

**Question:**

What are the prevalence of and risk factors for food insecurity among lesbian, gay, bisexual, queer, and similar (LGBQ+) veterans?

**Findings:**

In this cross-sectional study of 3 580 148 veterans receiving medical care through the Veterans Health Administration, LGBQ+ veterans experienced food insecurity at nearly 2.5 times the rate of heterosexual veterans. While individual risk factors for food insecurity were similar for veterans of all sexual orientations, LGBQ+ veterans experienced higher rates of some of these risk factors, including homelessness and several mental health and trauma-related comorbidities.

**Meaning:**

These findings suggest that LGBQ+ veterans are uniquely vulnerable to experiencing food insecurity, which often co-occurs with other social and medical risks; tailored strategies are needed to improve how food insecurity is identified and addressed in this population.

## Teaser

This cohort study examines associations of sociodemographic, clinical, and psychosocial characteristics of veterans identifying as lesbian, gay, bisexual, queer or another minority sexual orientation (LGBQ+) with positive screening results for food insecurity compared with other veterans.

## Introduction

Food insecurity, defined as limited or uncertain availability of nutritionally adequate and safe food, affects millions of US households^1^ and is associated with a myriad of adverse health outcomes.^2,3^ The Supplemental Nutrition Assistance Program (SNAP) helps mitigate food insecurity^4^; however, increased SNAP benefits distributed to households during the COVID-19 pandemic^5,6^ ended between 2021 and 2023 for all states,^7^ leaving millions of US residents with lower monthly SNAP allowances.^8,9^ The prevalence of food insecurity rose from 10.2% in 2021 to 12.8% in 2022 and to 13.5% in 2023,^1^ likely due in part to the end of several COVID-19 economic relief programs.^10^

Historically marginalized populations are at a particularly high risk for food insecurity; including racially and ethnically minoritized individuals^11,12,13^; women^12^; active-duty service members^14^; veterans,^15,16^ and sexual minority individuals including those identifying as lesbian, gay, bisexual, queer, and similar (LGBQ+).^17^ Material hardship and health disparities faced by sexual minority groups are well documented and part of a larger history of marginalization and structural inequities tied to stigma and discrimination.^18^ Compared with those identifying as heterosexual, individuals identifying as LGBQ+ are more likely to be unemployed, be uninsured, and have a lower health-related quality of life.^19^ Estimates of food insecurity among sexual minority groups in the US have ranged from 13%^19^ to as high as 27% among sexual minority women.^20^ Higher use of SNAP and emergency food assistance has been identified among sexual minority women compared with their heterosexual counterparts.^20^

While our understanding of food insecurity among LGBQ+ individuals and veterans overall has grown in recent years, there remains a dearth of research on food insecurity among sexual minority veterans, a population with unique social barriers and medical comorbidities. Due to the confluence of service-related health issues, adjustment to postdeployment civilian life, and potential employment challenges,^16^ the veteran population, especially post–Vietnam era veterans, are particularly vulnerable to experiencing material hardship.^21,22,23^ These challenges are compounded for LGBQ+ veterans, who may face both past and ongoing prejudice related to their sexual orientation.

Thousands of service members received dishonorable discharges under Don’t Ask, Don’t Tell, the US military’s 17-year ban on individuals openly serving as LGBQ+,^24^ preventing them from receiving service-connected medical, financial, and educational benefits. Since the ban’s repeal, veterans have been able to apply for a discharge upgrade, but many have not done so due to difficulties navigating the upgrade process and/or concerns about further discrimination or mistreatment.^25,26^

Over the past decade, providing high-quality, affirmative care for LGBQ+ veterans and collecting actionable data has become a top priority for the Veterans Health Administration (VHA), the country’s largest integrated health care system.^27^ The national VHA lesbian, gay, bisexual, transgender, queer, and similar (LGBTQ+) Health Program was created in 2012, followed by the 2016 rollout of LGBTQ+ veteran care coordinators at each VHA facility. A 2020 Government Accountability Office report recommended that the VHA standardize collection of sexual orientation and gender identity data in the medical record to better assess LGBTQ+ health outcomes.^28^ Following this, in September of 2022, the VHA launched a national sexual orientation clinical reminder to document veterans’ sexual orientation, assist clinicians in providing more tailored health care, and facilitate the identification of disparities among LGBQ+ veterans. The VHA has also invested heavily in improving veteran food security. In 2017, the VHA implemented a food insecurity clinical reminder,^29,30^ and in 2022 established the national VHA Food Security Office.

Little is known about the prevalence of or risk factors for food insecurity among LGBQ+ veterans. To address this gap, we examined (1) sociodemographic, clinical, and psychosocial characteristics of LGBQ+ veterans compared with heterosexual veterans and veterans with “don’t know” responses on the clinical reminder; and (2) characteristics of veterans with positive vs negative screening results for food insecurity among these 3 groups.

## Methods

This study was approved by the VHA Providence Healthcare System Institutional Review Board, which granted a waiver of informed consent because the research used deidentified medical record data. This study follows the Strengthening the Reporting of Observational Studies in Epidemiology (STROBE) reporting guideline.

### Data Source and Study Cohort

We used administrative data extracted from the VHA’s Corporate Data Warehouse. Our cohort included all veterans who were administered the VHA food insecurity clinical reminder between March 1, 2021 (when the VHA implemented updated food insecurity screening questions), and August 31, 2023, who were also administered the sexual orientation clinical reminder. A total of 1225 veterans declined, were unable to answer, or were inappropriately flagged as being eligible for food insecurity screening. An additional 322 374 veterans had “prefer not to answer” responses for their sexual orientation. Our final analytic sample included 3 580 148 veterans (Figure).

**Figure.**
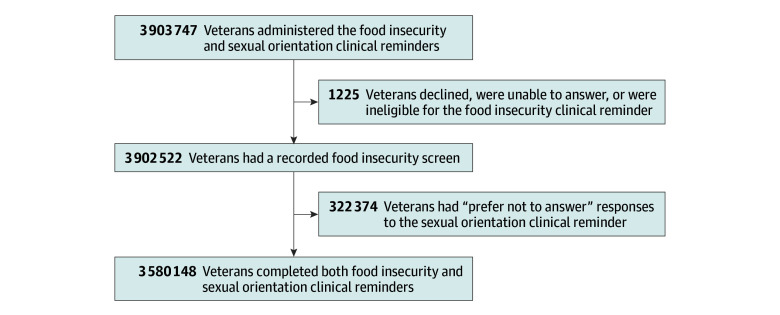
Flowchart of the Analytic Sample

### Measures

#### Food Insecurity

All veterans presenting for primary care and not residing in a long-term care facility are due to be screened annually for food insecurity.^29^ In March of 2021, VHA updated the food insecurity clinical reminder to the validated Hunger Vital Sign.^31^ The current clinical reminder asks, “Within the past 12 months you worried whether your food would run out before you got money to buy more” and “Within the past 12 months the food you bought just didn’t last and you didn’t have money to get more,” with response options of often true, sometimes true, or never true. Veterans who respond often true or sometimes true to one or both questions are considered food insecure and are offered a referral to a social worker and/or a dietitian. For veterans with more than 1 food insecurity screening during the study period, the index screening was defined as their first positive result, or if the veteran never had a positive result, their first screen.

#### Sexual Orientation

The sexual orientation clinical reminder, administered annually to all veterans by a clinical care team member, asks if they identify as “lesbian or gay,” “straight or heterosexual,” “bisexual,” “queer,” “don’t know,” “a sexual orientation not listed here: [open text],” or “prefer not to answer.” Veterans were stratified into groups of those who identified as heterosexual, those who responded “don’t know,” and those who identified as lesbian, gay, bisexual, queer, or a sexual orientation not listed (LGBQ+). Veterans who responded “prefer not to answer” were excluded from the analytic sample (Figure).

#### Sociodemographic Characteristics and Medical Outcomes

Sociodemographic characteristics, including birth sex, age at the time of screening, race, ethnicity, marital status, history of military sexual trauma (MST), and veteran enrollment priority status, were obtained from the medical record. Race and ethnicity were assessed because these have been associated with food security status in prior studies. Priority group refers to veterans’ eligibility for and cost share associated with Department of Veterans Affairs (VA) health benefits, as well as service-connected disability compensation. We collapsed the 8 VA enrollment priority groups into 3 categories^32^: (1) service connected (VA groups 1-4), (2) non–service connected and low income (VA group 5), and (3) non–service connected and not low income (VA groups 6-8). We used *International Statistical Classification of Diseases, Tenth Revision* (*ICD-10*), codes to identify the following diagnoses within 2 years prior to food insecurity screening: depression, anxiety, suicidality (suicidal ideation or attempt), posttraumatic stress disorder (PTSD; ever diagnosed), and substance use disorder (SUD). Veterans living in rural areas were identified using a standardized VA definition based on rural-urban commuting area codes. We defined homelessness or housing instability as having a positive result for the homelessness clinical reminder screen (which assesses both recent and risk of near-future homelessness or housing instability^33^) in the prior 18 months, and/or an *ICD-10* code indicating homelessness or housing instability in the prior 2 years. Veterans with a history of interpersonal violence (IPV) were identified from a positive result for the IPV clinical reminder screen in the prior 18 months.

### Statistical Analysis

Standard descriptive statistics (frequencies and percentages) were calculated for all variables. We compared variables of interest stratified by screening status. We used χ^2^ tests for categorical variables and 2-tailed *t* tests for continuous variables. Two-sided *P* < .05 indicated statistical significance. We used R, version 4.4.1 (R Program for Statistical Computing) for statistical analysis.

## Results

Of 3 580 148 veterans screened, the mean (SD) age was 61.6 (0.4) years; 387 641 (10.8%) were assigned female sex at birth, and 3 192 507 (89.2%) were assigned male sex at birth. In terms of race and ethnicity, 33 755 (0.9%) were American Indian or Alaska Native; 53 446 (1.5%) were Asian; 661 980 (18.5%) were Black; 288 074 (8.0%) were Hispanic; 36 958 (1.0%) were Native Hawaiian or Other Pacific Islander; 2 248 424 (62.8%) were White; and 257 511 (7.2%) were missing data (Table 1). A total of 83 292 veterans (2.3%) identified as LGBQ+, and 10 183 (0.3%) had “don’t know” responses to the sexual orientation clinical reminder. Compared with heterosexual veterans, LGBQ+ veterans were overall younger (mean [SD] age, 50.2 [17.8] vs 61.9 [16.6] years) and more likely to have female sex assigned at birth (36 174 [44.1%] vs 349 037 [10.0%]), be part of a minoritized racial or ethnic group (28 872 [34.7%] vs 1 041 632 [29.8%]), and be unmarried or unpartnered (57 548 [69.1%] vs 1 385 158 [39.7%]). LGBQ+ veterans (8602 [10.3%]) and those with “don’t know” responses (1081 [10.6%]) were nearly twice as likely as heterosexual veterans (188 586 [5.4%]) to have experienced recent homelessness or housing instability. Compared with heterosexual veterans, LGBQ+ veterans were also more likely to have anxiety (6430 [7.7%] vs 150 304 [4.3%]), depression (25 901 [31.1%] vs 673 507 [19.3%]), PTSD (35 177 [42.2%] vs 1 053 524 [30.2%]), and SUD (10 952 [13.1%] vs 314 310 [9.0%]) and were more than twice as likely to experience suicidality (3030 [3.6%] vs 47 297 [1.4%]). Nearly one-fourth of LGBQ+ veterans (20 030 [24.0%]) had experienced MST compared with 187 588 heterosexual veterans (5.4%), and LGBQ+ veterans were almost twice as likely as heterosexual veterans to have a recent history of IPV (2185 [2.6%] vs 48 594 [1.4%]).

**Table 1.** Characteristics of Veterans Screened Using the Food Insecurity and Sexual Orientation Clinical Reminders, March 2021 to August 2023

Characteristics	Veterans group, No. (%)				*P* value^a^
	Overall (N = 3 580 148)	LGBQ+ (n = 83 292)	Heterosexual (n = 3 486 673)	“Don’t know” response (n = 10 183)	
Age, mean (SD), y	61.6 (16.7)	50.2 (17.8)	61.9 (16.6)	60.9 (17.7)	NA
Age range, y					
18-34	311 109 (8.7)	22 201 (26.7)	287 776 (8.3)	1132 (11.1)	<.001
35-44	407 847 (11.4)	15 483 (18.6)	391 201 (11.2)	1163 (11.4)	<.001
45-54	452 197 (12.6)	10 830 (13.0)	440 193 (12.6)	1174 (11.5)	<.001
55-64	638 289 (17.8)	14 471 (17.4)	621 984 (17.8)	1834 (18.0)	.002
≥65	1 770 706 (49.5)	20 307 (24.4)	1 745 519 (50.1)	4880 (47.9)	<.001
Sex assigned at birth					
Female	387 641 (10.8)	36 174 (44.1)	349 037 (10.0)	1843 (18.1)	<.001
Male	3 192 507 (89.2)	45 894 (55.9)	3 137 636 (90.0)	8340 (81.9)	<.001
Race and ethnicity					
American Indian or Alaska Native	33 755 (0.9)	1059 (1.3)	32 594 (0.9)	102 (1.0)	<.001
Asian	53 446 (1.5)	1437 (1.7)	51 787 (1.5)	222 (2.2)	<.001
Black, non-Hispanic	661 980 (18.5)	16 731 (20.1)	642 745 (18.4)	2504 (24.6)	<.001
Hispanic	288 074 (8.0)	8660 (10.4)	278 695 (8.0)	719 (7.1)	<.001
Native Hawaiian or Other Pacific Islander	36 958 (1.0)	985 (1.2)	35 811 (1.0)	162 (1.6)	<.001
White, non-Hispanic	2 248 424 (62.8)	48 186 (57.9)	2 194 487 (62.9)	5751 (56.5)	<.001
Missing	257 511 (7.2)	6234 (7.5)	250 554 (7.2)	723 (7.1)	.004
Marital status					
Divorced, separated, or widowed	942 919 (26.3)	22 366 (26.9)	917 169 (26.3)	3384 (33.2)	<.001
Married or partnered	1 990 091 (55.6)	23 126 (27.8)	1 963 068 (56.3)	3897 (38.3)	<.001
Single, never married	505 650 (14.1)	35 182 (42.2)	467 989 (13.4)	2479 (24.3)	<.001
Missing	141 488 (4.0)	2618 (3.1)	138 447 (4.0)	423 (4.2)	<.001
Enrollment priority^b^					
SC disability^c^	2 372 884 (66.3)	57 206 (68.7)	2 309 056 (66.2)	6622 (65.0)	<.001
Non-SC and low income^d^	534 269 (14.9)	13 690 (16.4)	518 629 (14.9)	1950 (19.1)	<.001
Non-SC and not low income^e^	664 340 (18.6)	11 866 (14.2)	650 890 (18.7)	1584 (15.6)	<.001
Missing	8655 (0.2)	530 (0.6)	8098 (0.2)	27 (0.3)	<.001
Homelessness or housing instability	198 269 (5.5)	8602 (10.3)	188 586 (5.4)	1081 (10.6)	<.001
Rurality	1 087 793 (30.4)	15 363 (18.4)	1 070 176 (30.7)	2254 (22.1)	<.001
Anxiety	157 288 (4.4)	6430 (7.7)	150 304 (4.3)	554 (5.4)	<.001
Depression	701 899 (19.6)	25 901 (31.1)	673 507 (19.3)	2491 (24.5)	<.001
Suicidality	50 585 (1.4)	3030 (3.6)	47 297 (1.4)	258 (2.5)	<.001
Posttraumatic stress disorder	1 092 241 (30.5)	35 177 (42.2)	1 053 524 (30.2)	3540 (34.8)	<.001
Substance use disorder	326 483 (9.1)	10 952 (13.1)	314 310 (9.0)	1221 (12.0)	<.001
History of military sexual trauma	208 714 (5.8)	20 030 (24.0)	187 588 (5.4)	1096 (10.8)	<.001
Intimate partner violence	50 899 (1.4)	2185 (2.6)	48 594 (1.4)	120 (1.2)	<.001

Abbreviations: LGBQ+, lesbian, gay, bisexual, queer, and similar; NA, not applicable; SC, service connected.

^a^ Estimated from χ^2^ tests.

^b^ Enrollment priority determines US veterans’ eligibility for and cost share associated with Veterans Health Administration (VHA) health benefits.

^c^ SC disability provides a monetary benefit paid to veterans who are determined by Department of Veterans Affairs (VA) to be disabled by an injury or illness that was incurred or aggravated during active military service.

^d^ Includes non-SC veterans who have income below the VA-administered means test cutoff.

^e^ Includes non-SC veterans who have income above the VA-administered means test cutoff.

Overall, 96 413 veterans (2.7%) had positive screen results for food insecurity (Table 2). LGBQ+ veterans (5352 [6.4%]) and veterans with “don’t know” responses (635 [6.2%]) had nearly 2.5 times the rate of positive food insecurity screen results compared with heterosexual veterans (90 426 [2.6%]). Risk factors for food insecurity were overall similar among LGBQ+ veterans, heterosexual veterans, and those with “don’t know” responses. Regardless of sexual orientation, all veterans experiencing food insecurity were more likely to be younger, to be unmarried or unpartnered, to have female sex assigned at birth, and to identify as American Indian or Alaska Native, Black, Hispanic, or Native Hawaiian or Other Pacific Islander. Veterans experiencing food insecurity across all sexual orientations were also more likely to have anxiety, depression, suicidality, PTSD, SUD, and a history of MST and IPV. More than half of veterans with food insecurity across sexual orientation groups had experienced recent housing insecurity or homelessness (54.6% for LGBQ+ veterans vs 54.3% for heterosexual veterans). LGBQ+ veterans had significantly higher rates of several risk factors compared with heterosexual veterans, including being younger than 45 years (45.3% vs 19.5%), female sex assigned at birth (44.1% vs 10.0%), being in a minoritized racial or ethnic group (34.7% vs 29.8%), unmarried or unpartnered status (69.1% vs 39.7% and 57.6%, respectively), low income (16.4% vs 14.9%), homelessness or housing instability (10.3% vs 5.4%), anxiety (7.7% vs 4.3%), depression (31.1% vs 19.3%), suicidality (3.6% vs 1.4%), PTSD (42.2% vs 30.2%), SUD (13.1% vs 9.0%), MST (24.0% vs 5.4%), and recent IPV (2.6% vs 1.4%).

**Table 2.** Characteristics of Veterans Food Insecurity Positive and Negative Food Insecurity Screen Results by Recorded Sexual Orientation, March 2021 to August 2023

Characteristic	LGBQ+ veterans, No. (%)			Heterosexual veterans, No. (%)			Veterans with “don’t know” responses, No. (%)		
	No food insecurity (n = 77 940 [93.6%])	Food insecurity (n = 5352 [6.4%])	*P* value^a^	No food insecurity (n = 3 396 247 [97.4%])	Food insecurity (n = 90 426 [2.6%])	*P* value^a^	No food insecurity (n = 9548 [93.8%])	Food insecurity (n = 635 [6.2%])	*P* value^a^
Age, mean (SD), y	50.7 (17.9)	43.4 (15.1)	NA	62.1 (16.6)	54 (15.3)	NA	61.4 (17.6)	53.1 (16.4)	NA
Age range, y									
18-34	20 171 (25.9)	2030 (37.9)	<.001	274 489 (8.1)	13 287 (14.7)	<.001	1011 (10.6)	121 (19.1)	<.001
35-44	14 278 (18.3)	1205 (22.5)	<.001	375 997 (11.1)	15 204 (16.8)	<.001	1066 (11.2)	97 (15.3)	.002
45-54	10 140 (13.0)	690 (12.9)	.80	426 254 (12.6)	13 939 (15.4)	<.001	1085 (11.4)	89 (14.0)	.05
55-64	13 594 (17.4)	877 (16.4)	.05	598 479 (17.6)	23 505 (26.0)	<.001	1683 (17.6)	151 (23.8)	<.001
≥65	19 757 (25.3)	550 (10.3)	<.001	1 721 028 (50.7)	24 491 (27.1)	<.001	4703 (49.3)	177 (27.9)	<.001
Sex assigned at birth									
Female	34 231 (43.9)	2530 (47.3)	<.001	335 667 (9.9)	13 370 (14.8)	<.001	1702 (17.8)	141 (22.2)	.006
Male	43 709 (56.1)	2822 (52.7)	<.001	3 060 580 (90.1)	77 056 (85.2)	<.001	7846 (82.2)	494 (77.8)	.006
Race and ethnicity									
American Indian or Alaska Native	949 (1.2)	110 (2.1)	<.001	31 055 (0.9)	1539 (1.7)	<.001	85 (0.9)	17 (2.7)	<.001
Asian	1368 (1.8)	69 (1.3)	.04	50 661 (1.5)	1126 (1.2)	<.001	212 (2.2)	NA^b^	.30
Black, non-Hispanic	15 147 (19.4)	1584 (29.6)	<.001	612 399 (18.0)	30 346 (33.6)	<.001	2278 (23.9)	226 (35.6)	<.001
Hispanic	8084 (10.4)	576 (10.8)	.40	270 322 (8.0)	8373 (9.3)	<.001	657 (6.9)	62 (9.8)	.008
Native Hawaiian or Other Pacific Islander	917 (1.2)	68 (1.3)	.60	34 653 (1.0)	1158 (1.3)	<.001	151 (1.6)	11 (1.7)	.90
White, non-Hispanic	45 703 (58.6)	2483 (46.4)	<.001	2 153 338 (63.4)	41 149 (45.5)	<.001	5497 (57.6)	254 (40.0)	<.001
Missing	5772 (7.4)	462 (8.6)	.001	243 819 (7.2)	6735 (7.4)	.002	668 (7.0)	55 (8.7)	.10
Marital status									
Divorced, separated, or widowed	20 693 (26.5)	1673 (31.3)	<.001	878 679 (25.9)	38 490 (42.6)	<.001	3114 (32.6)	270 (42.5)	<.001
Married or partnered	22 086 (28.3)	1040 (19.4)	<.001	1 936 565 (57.0)	26 503 (29.3)	<.001	3757 (39.3)	140 (22.0)	<.001
Single, never married	32 674 (41.9)	2508 (46.9)	<.001	445 302 (13.1)	22 687 (25.1)	<.001	2273 (23.8)	206 (32.4)	<.001
Missing	2487 (3.2)	131 (2.4)	.003	135 701 (4.0)	2746 (3.0)	<.001	404 (4.2)	19 (3.0)	.20
Enrollment priority^c^									
SC disability^d^	53 570 (68.7)	3636 (67.9)	.20	2 254 970 (66.4)	54 086 (59.8)	<.001	6219 (65.1)	403 (63.5)	.4
Non-SC and low income^e^	12 446 (16.0)	1244 (23.2)	<.001	491 276 (14.5)	27 353 (30.2)	<.001	1764 (18.5)	186 (29.3)	<.001
Non-SC and not low income^f^	11 452 (14.7)	414 (7.7)	<.001	642 400 (18.9)	8490 (9.4)	<.001	1542 (16.1)	42 (6.6)	<.001
Missing	472 (0.6)	58 (1.1)	<.001	7601 (0.2)	497 (0.5)	<.001	23 (0.2)	NA^b^	.10
Homelessness or housing instability	5680 (7.3)	2922 (54.6)	<.001	139 453 (4.1)	49 133 (54.3)	<.001	743 (7.8)	338 (53.2)	<.001
Rurality	14 624 (18.8)	739 (13.8)	<.001	1 051 082 (30.9)	19 094 (21.1)	<.001	2156 (22.6)	98 (15.4)	<.001
Anxiety	5856 (7.5)	574 (10.7)	<.001	142 785 (4.2)	7519 (8.3)	<.001	501 (5.2)	53 (8.3)	<.001
Depression	23 615 (30.3)	2286 (42.7)	<.001	640 570 (18.9)	32 937 (36.4)	<.001	2249 (23.6)	242 (38.1)	<.001
Suicidality	2450 (3.1)	580 (10.8)	<.001	40 313 (1.2)	6984 (7.7)	<.001	194 (2.0)	64 (10.1)	<.001
Posttraumatic stress disorder	31 953 (41.0)	3224 (60.2)	<.001	1 010 012 (29.7)	43 512 (48.1)	<.001	3220 (33.7)	320 (50.4)	<.001
Substance use disorder	9568 (12.3)	1384 (25.9)	<.001	290 060 (8.5)	24 250 (26.8)	<.001	1031 (10.8)	190 (29.9)	<.001
History of military sexual trauma	17 981 (23.1)	2049 (38.3)	<.001	175 368 (5.2)	12 220 (13.5)	<.001	973 (10.2)	123 (19.4)	<.001
Intimate partner violence	1775 (2.3)	410 (7.7)	<.001	44 425 (1.3)	4169 (4.6)	<.001	99 (1.0)	21 (3.3)	<.001

Abbreviations: LGBQ+, lesbian, gay, bisexual, queer, and similar; NA, not applicable; SC, service connected.

^a^ Estimated from χ^2^ tests.

^b^ Sample size of 10 or less.

^c^ Enrollment priority determines US veterans’ eligibility for and cost share associated with Veterans Health Administration health benefits.

^d^ SC disability provides a monetary benefit paid to veterans who are determined by the Department of Veterans Affairs (VA) to be disabled by an injury or illness that was incurred or aggravated during active military service.

^e^ Includes non-SC veterans who have income below the VA-administered means test cutoff.

^f^ Includes non-SC veterans who have income above the VA-administered means test cutoff.

## Discussion

Among veterans receiving VHA health care between March of 2021 and August of 2023, LGBQ+ veterans and those with “don’t know” responses regarding their sexual orientation experienced food insecurity at nearly 2.5 times the rate of heterosexual veterans. While individual risk factors for food insecurity were similar for veterans of all sexual orientations, LGBQ+ veterans had higher rates of several risk factors compared with heterosexual veterans, including age younger than 45 years, female sex assigned at birth, being in a minoritized racial or ethnic group, unmarried or unpartnered status, low income, homelessness or housing instability, anxiety, depression, suicidality, PTSD, SUD, MST, and IPV. Our findings demonstrate a higher likelihood of food insecurity and specific food insecurity–related risks for LGBQ+ veterans, contributing to increased vulnerability and burden among this population.

Our study is the first, to our knowledge, to examine food insecurity among LGBQ+ veterans. A previous analysis of the Federal Reserve’s 2019 Survey of Household Economics and Decision-Making by the Center for American Progress^34^ found that LGBQ+ veterans were twice as likely as non–LGBQ+ veterans to report using SNAP in the past 12 months and to be receiving unemployment benefits. They did not, however, explicitly examine reported food insecurity.

Our findings of risk factors for food insecurity in the overall veteran population were consistent with those of prior studies, including younger age,^16,22,29,35^ female sex assigned at birth,^16,29,36^ being in a minoritized racial or ethnic group,^15,29,36,37,38^ low income,^15,16,29,35,36,37,38^ unmarried or unpartnered status,^16,29,35,36^ housing instability or homelessness,^15,29,39^ depression,^15,29,35,36,37,38,40,41^ anxiety,^41^ PTSD,^29,37,38^ MST,^29,42^ suicidality,^38,40,42^ and SUD.^29,38^ We also found that IPV was a risk factor for food insecurity, a finding not previously reported in the veteran population but consistent with work conducted outside the VHA.^43,44^ Importantly, experiences of food insecurity may also contribute to some of these risks, many of which are bidirectional.^30^

Our findings on higher rates of mental health conditions among LGBQ+ veterans were consistent with those of prior studies^42,45,46,47^ showing LGBQ+ veterans are significantly more likely to experience anxiety, depression, PTSD, and suicidality than heterosexual veterans. Increased burden of mental health disorders among sexual minority individuals is well documented.^46,48,49^ Meyer’s theory of minority stress^18^ describes this higher prevalence of mental health conditions among sexual minority groups as a byproduct of exposure to identity-related stressors, including prejudice and discrimination and hiding one’s sexual minority identity. Prior work^50^ reveals LGBQ+ veterans report experiences of discrimination from both health care professionals and fellow veterans. Studies have also linked symptoms of depression and PTSD among LGBQ+ veterans to the stressors of having to conceal their sexual orientation during service.^51^

Like previous work,^42,51^ LGBQ+ veterans in our cohort also experienced higher rates of SUD, likely related to the highly comorbid nature of SUD and mental health disorders.^52,53,54^ Our finding that LGBQ+ veterans were more likely than heterosexual counterparts to experience MST and IPV is also consistent with prior work.^42,55,56^

LGBQ+ veterans were nearly twice as likely as heterosexual veterans to have experienced recent homelessness or housing instability, paralleling findings that sexual minority adults are more likely to have experienced homelessness at some point in their life compared with the general population.^57^ Risk factors for homelessness among veterans, including a low income, MST, mental health conditions, and SUD, were all more prevalent among LGBQ+ veterans in our sample.

Our findings that LGBQ+ veterans had a higher prevalence of mental health and trauma-related comorbidities as well as unmet social needs, each of which are risk factors for food insecurity, highlight the distinct and complex challenges faced by LGBQ+ veterans. They also underscore the intersecting, often compounding effect that structural inequities have on individuals’ health and well-being.^58^

Though they comprised a small percentage of our cohort, veterans with “don’t know” responses to the sexual orientation clinical reminder were analyzed as a separate group due to identifying as neither heterosexual nor explicitly LGBQ+. Notably, veterans with “don’t know” responses experienced similar rates of food insecurity as LGBQ+ veterans. They also experienced rates of housing insecurity and SUD that were more similar to LGBQ+ veterans than heterosexual veterans, and higher rates of mental health conditions and MST compared with heterosexual veterans. However, in terms of age and sex assigned at birth, veterans with “don’t know” responses were more similar to heterosexual veterans than to LGBQ+ veterans. Future work is needed to better understand this population and their risk factors for and experiences of food insecurity and related material hardship.

Together our findings highlight (1) the importance of continuing to collect sexual orientation data on veterans receiving VHA care to elucidate health care inequities and target tailored assistance; (2) the increased risk both for food insecurity and a number of associated mental health and trauma-related risks faced by LGBQ+ veterans; and (3) the need for continued attention to address veteran food insecurity, especially in minoritized populations, including LGBQ+ veterans. Our findings can help clinicians and health systems understand risks for food insecurity, including numerous comorbidities that are more prevalent among LGBQ+ veterans, and reinforce the need for clinicians to ask about sexual orientation as a health-related characteristic. The strong association between food insecurity and MST, IPV, and PTSD underscores the continued need for trauma-informed and culturally competent care, particularly among LGBQ+ veterans who bear a high trauma burden. Existing VHA programs, such as Pride in All Who Served, a group health education program, has shown strong success among participating veterans, including improvements in acceptance concerns, identity uncertainty,^59^ and likelihood of future suicide attempts.^60^

Future work is needed to better understand the potential role of more frequent and different methods of food insecurity screening among high-risk populations, including LGBQ+ veterans. Additionally, while the VHA has shown some expansion in settings where screening is conducted, the VHA should consider formalizing routine screening beyond primary care to include mental health, women’s health, LGBTQ+ health programs, and other specialty care clinics. Recognizing that individuals experiencing food insecurity are also at increased risk for use of acute care services,^61,62,63^ several other health systems have also adopted routine screening for food insecurity in the emergency department and/or inpatient settings.^64,65,66^

While identifying veterans experiencing food insecurity is a critical first step, efforts are also needed to ensure veterans can access needed resources to support both short- and long-term food security. According to a 2021 study,^36^ fewer than one-third of veterans with a low income participate in SNAP. Closing the SNAP eligibility-enrollment gap through increased outreach and assistance continues to be a key priority within the VA. Additionally, 74 VA medical centers have onsite or mobile food pantries and/or food boxes. Other initiatives being piloted include produce prescription programs and the establishment of “food hubs” designed to provide wraparound services for veterans experiencing food insecurity, including same-day access to social workers and dietitians, in addition to provision of food items. Food hubs can also serve as an entry point into VHA care for those not already enrolled. The VHA additionally has a national quality improvement initiative focused on screening for, assessing, and addressing veterans’ social needs more broadly, which is critical given the interplay of food insecurity with other unmet needs.^67,68^

### Strengths and Limitations

We were able to evaluate a national cohort of 3.5 million veterans who were administered both the food insecurity and sexual orientation clinical reminders, which is a substantial strength of this study. This study also has several limitations. Responses to the sexual orientation and food insecurity clinical reminders may have been influenced by perceived stigma, veterans’ trust in the individual administering screening, and/or staff comfort asking questions related to sexual orientation and food security. Additionally, as responses are administered and recorded by staff, in certain instances staff may have completed reminders based on assumptions of veterans’ sexual orientation and/or food security status rather than asking and recording veteran-reported responses. Future work should consider the feasibility of veteran self-administered screening for clinical reminders, particularly considering prior findings that patients often prefer self-administered over staff-administered screening for sensitive topics and have higher rates of disclosure for food insecurity in clinical settings when questions are self-administered.^69,70,71^ Finally, analyses were limited to veterans who presented for VHA care and were screened during our study period, so findings may not be generalizable to all US veterans.

## Conclusions

In this cohort study of veterans receiving VA care, we found LGBQ+ veterans were uniquely vulnerable to experiencing food insecurity. Tailored strategies are needed to improve how food insecurity is identified and addressed in this population, with particular attention to the role of intersecting risks factors, including high trauma burden and mental health comorbidities.

## Notes

Supplement.
Data Sharing Statement
