# Supplementary material for: Food Insecurity Among LGBQ+ Veterans
Source: JAMA Netw Open. 2024 Nov 4;7(11):e2442979. doi: 10.1001/jamanetworkopen.2024.42979 (PMC11536314; doi:10.1001/jamanetworkopen.2024.42979)
Supplement: Supplement. — Data Sharing Statement [file jamanetwopen-e2442979-s001.pdf]

Data Sharing Statement

Haigh. Food Insecurity Among LGBQ+ Veterans. *JAMA Netw Open*. Published November 04, 2024. doi:10.1001/jamanetworkopen.2024.42979

Data
Data available: No

Additional Information
Explanation for why data not available: Access to VA administrative and electronic health record data is restricted, and per VA policy it is not possible to make this dataset publicly available.
